# Unexpected Airway Collapse: A Rare Case of Spontaneous Postoperative Tracheobronchomalacia in the Absence of Identifiable Risk Factors

**DOI:** 10.7759/cureus.59078

**Published:** 2024-04-26

**Authors:** Will S Roberts, Shawn Price, Brendan P Chernicki, Justin Reidy, Tammy L Birbeck

**Affiliations:** 1 Medical School, Nova Southeastern University Dr. Kiran C. Patel College Of Osteopathic Medicine, Clearwater, USA; 2 Osteopathic Medicine, Dr. Kiran C. Patel College of Osteopathic Medicine, Davie, USA; 3 Medical School, Nova Southeastern University Dr. Kiran C. Patel College of Osteopathic Medicine, Davie, USA; 4 Medical School, Nova Southeastern University Dr. Kiran C. Patel College of Osteopathic Medicine, Clearwater, USA; 5 Gynecologic Surgery, Gulf Women’s Center for Health and Surgery, Englewood, USA

**Keywords:** intubation complication, flexible bronchoscopy, excessive dynamic airway collapse, general anesthesiology, tracheobronchomalacia, surgical outcome, postoperative pulmonary complication

## Abstract

We report the case of a 53-year-old female who developed tracheobronchomalacia immediately following an uncomplicated robotic hysterectomy with bilateral salpingo-oophorectomy to treat postmenopausal bleeding. Induction of anesthesia was notable for moderately difficult intubation, managed with applied cricothyroid pressure and a small 6.5 endotracheal tube placement via GlideScope. The surgical course was uneventful. The patient remained intubated in the post-anesthesia care unit but was not providing end-tidal volumes. Attempts to replace the endotracheal tube with a larger tube were unsuccessful and the patient was temporarily unable to ventilate. Rapid troubleshooting discovered that a laryngeal mask airway (LMA) could sufficiently ventilate the patient. An otolaryngologist was able to perform direct bronchoscopy, which revealed more than 50% dynamic anterior-posterior collapse of the trachea and bronchi. The patient was subsequently awakened from anesthesia and monitored in the intensive care unit, ventilating with an LMA. After a couple of hours, it was determined that the patient’s airway was protected, and the LMA was removed.

## Introduction

Tracheobronchomalacia (TBM) is an uncommon complication of various chronic pulmonary diseases, most notably chronic bronchitis [1]. It is defined as a dynamic collapse of the trachea and bronchi on exhalation that occludes at least 50% of the airway lumen [2]. Clinically, there are two forms of TBM: primary and secondary [3]. Primary TBM is seen in neonates and is the result of a congenital defect leading to structural impairment or compression of the airway structures [3]. Secondary TBM, also known as acquired TBM, is commonly the result of repetitive trauma and degradation of the structural support of the trachea and bronchi, precipitating their collapse [4]. The sequelae of TBM can be fatal, as the inability to sufficiently exhale leads to the retention of carbon dioxide and end-organ damage [5].

The precise incidence of acquired TBM in the general population is difficult to assess because mild cases are commonly asymptomatic [6]. However, it is almost exclusively associated with other chronic pulmonary diseases such as chronic obstructive pulmonary disease (COPD), asthma, emphysema, and chronic bronchitis [7]. It is theorized that these diseases place significant stress on the tracheal cartilage rings over time, weakening their structural integrity [7]. There have been very few documented cases of TBM arising in the absence of identified risk factors.

## Case presentation

Preoperative course

A 53-year-old female with a prior medical history of hyperlipidemia treated with atorvastatin 10 mg presented to a small community hospital for elective robotic hysterectomy with bilateral salpingo-oophorectomy for definitive treatment of abnormal postmenopausal bleeding. Prior surgical history was notable for a right parotidectomy to treat acinic cell carcinoma seven years ago, which proceeded without complication under general anesthesia. The patient elected to undergo surgical resection as monotherapy for her acinic cell carcinoma and did not receive any chemotherapy or radiation. Preoperative physical examination was unremarkable. Preoperative vital signs were blood pressure (BP) of 110/57 mmHg, heart rate (HR) of 78 beats per minute, respiratory rate (RR) of 20 respirations per minute, oxygen saturation of 99% on room air, temperature of 97.8°F, and body mass index (BMI) of 25.4 kg/m^2^. The patient was classified as an American Society of Anesthesiologists II and Mallampati II by the anesthesiologist.

Induction

The patient was transported to the operating room and administered midazolam 2 mg intravenously (IV) for anxiolysis immediately upon arrival. Standard monitoring of BP, HR, ventilation, oxygenation, and temperature was established. The patient was then induced with 200 mg of propofol. Following induction, fentanyl 50 µg, cefoxitin 2 g, and rocuronium 70 mg were administered. Intubation was difficult due to significant anterior deviation of the trachea but was managed with the application of cricothyroid pressure and the utilization of a small 6.5 endotracheal (ET) tube placed with GlideScope. Intubation was achieved on the second attempt once the aforementioned assistance was applied and the 7.5 ET tube was switched to a 6.5 ET tube. The ET tube was placed at a depth of 21 cm.

Perioperative course

The perioperative period was uneventful. The abdominal cavity was insufflated with carbon dioxide and five robotic ports were established. The uterus was disconnected from the round and infundibulopelvic ligaments and removed vaginally along with the fallopian tubes and ovaries. The patient remained stable throughout the procedure without any significant alterations in vitals. Anesthesia was maintained with sevoflurane.

Emergence

Upon completion of the procedure, the patient was taking significantly longer than expected to emerge from anesthesia. Neostigmine 4 mg and glycopyrrolate 0.8 mg were administered to reverse paralysis. Due to delayed emergence, the patient was transported to the post-anesthesia care unit (PACU) while still intubated. The patient remained in the operating room (OR) for approximately 12 minutes before the PACU transfer. The reversal was monitored quantitatively via the train-of-four (TOF) ratio. The TOF was recorded at 0.7 upon exiting the OR. Upon arrival at the PACU, the patient’s vitals were stable and recorded at a BP of 128/58 mmHg, HR of 97 beats per minute, SpO_2_ of 97%, and RR of 20 respirations per minute. Initial PACU ventilatory settings were FiO_2_ of 97%, RR of 17 respirations per minute, tidal volume (TV) of 520 mL, and positive end-expiratory pressure (PEEP) of 5.4 cmH_2_O. However, the patient stopped producing end-tidal carbon dioxide volumes, despite seemingly regular inspiratory effort shortly after arrival. Upon initial decompensation, monitors were readjusted, pulses were checked, and cardiac sounds were auscultated, all of which did not reveal any abnormalities. At this point, it was hypothesized that the etiology was secondary to the small ET tube placed. The decision was made to replace the small 6.5 ET and re-intubate with a larger 8.5 ET to assist with ventilation. The ET tube remained at 21 cm until the patient was extubated. Upon extubation, the patient immediately stopped ventilating and began desaturating and the larger ET tube could not be placed. Re-intubation was attempted without redosing paralytics or utilization of a tube exchanger as it was not rapidly available. Further, attempts at replacing the small 6.5 ET were also unsuccessful. At this point, the patient’s oxygen saturation was rapidly decreasing and plummeted as low as 79%. It was unknown why the ET tubes would not pass into the trachea, so a laryngeal mask airway (LMA) was placed, allowing for patient ventilation. Additionally, the patient was administered racemic epinephrine 2.25% via respiratory therapy to promote bronchodilation.

**Figure 1:**
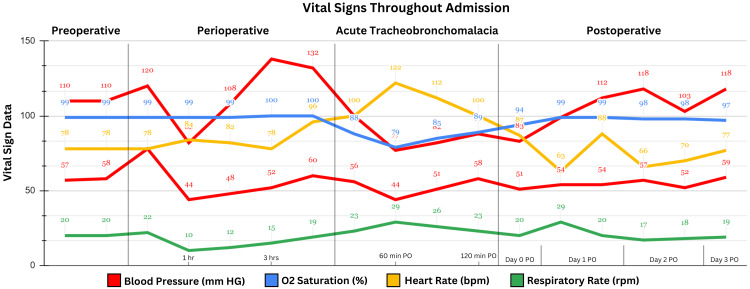
Vital signs throughout admission. The figure depicts the blood pressure (mmHg), oxygen saturation (%), heart rate (beats per minute), and respiratory rate (respirations per minute) from the preoperative to postoperative periods.

At this point, it was clear that there was an underlying airway issue preventing the intubation of the patient, and a local otolaryngologist was emergently consulted. As this case occurred in a small community hospital, there were no otolaryngologists, intensivists, or pulmonologists on staff to perform emergent bronchoscopies. Therefore, it took approximately one hour for the otolaryngologist to arrive. Per their request, the patient was prepped and transported back to the OR. During this time, the patient was slowly improving on LMA ventilation. The ventilatory parameters on LMA that allowed patient stabilization were an FiO_2_ of 100%, RR of 18 respirations per minute, TV of 430 mL, and PEEP of 15 cmH_2_O. Tracheostomy was considered should the patient desaturate further. Upon arrival of the otolaryngologist, the patient was re-induced with a second 200 mg of propofol. The LMA was removed immediately before bronchoscopy to allow for scope passage. A bronchoscope was inserted into the right nostril and passed through the middle meatus, revealing a normal nasopharynx. The bronchoscope was then passed toward the hypopharynx, which required a jaw thrust technique to pass the bronchoscope through due to significant narrowing. The epiglottis, false vocal cords, and true vocal cords were normal. Upon passing the bronchoscope through the glottis, greater than 50% anterior-posterior tracheal collapse was noted throughout the trachea and bilateral bronchi. The bronchoscope was removed and the patient was ventilated on LMA until she emerged from anesthesia. At this point, the LMA was removed and the patient protected their own airway. The vital signs from the preoperative to postoperative periods are presented in Figure 1.

Postoperative course

Upon removal of the LMA, the patient was transported to the intensive care unit (ICU) for close monitoring. Her vitals on ICU admission were a BP of 95/53 mmHg, HR of 97 beats per minute, RR of 20 respirations per minute, oxygen saturation of 95% on a 2 L nasal cannula, and temperature of 97°F. She remained stable throughout admission. The following morning, rales were auscultated in the right lower lung field and a chest X-ray revealed a subtle consolidation, consistent with atelectasis. The primary symptoms experienced by the patient were hoarseness and pain with speaking. She denied any symptoms of infection. The patient was started on prophylactic piperacillin-tazobactam 3.375 g Q8H IV. She continued to improve daily and was discharged two days later.

Clinical follow-up

The patient was seen in the clinic eight days postoperatively. Her only reported symptoms were continued hoarseness and pain with speaking, but she maintained that they both improved from her admission. She denied any relapse of symptoms associated with TBM. The current plan established by the otolaryngologist is no further management, as long as she remains asymptomatic.

## Discussion

**Figure 2:**
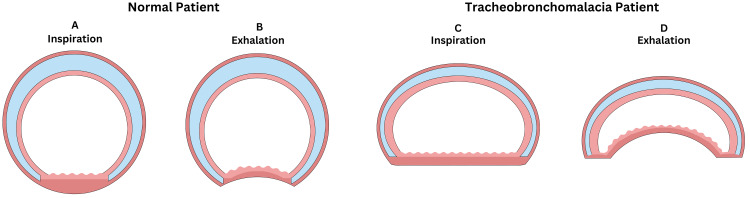
Overview of tracheobronchomalacia. The figure depicts the basic pathophysiology of tracheobronchomalacia compared to a normal patient during both inspiration and exhalation. A: Fully patent airway on inhalation in a normal patient. B: Fully patent airway on exhalation in a normal patient. C: Misshapen but fully patent airway on inhalation in a tracheobronchomalacia patient. D: Significant tracheal collapse and >50% airway occlusion on exhalation in a tracheobronchomalacia patient. Image credits: Will S. Roberts.

TBM is a well-documented sequelae of certain chronic pulmonary diseases, most notably chronic bronchitis [1]. It is theorized that the repetitive microtrauma to the tracheal cartilage and trachealis muscle can precipitate TBM [7]. The pathologic foundation of this condition lies in the anterior herniation of the trachealis muscle into the airway lumen following this cartilaginous trauma [7] (Figure 2). However, there have been very few reported cases of spontaneous TBM in the absence of any underlying pulmonary disease. Further, the reported patient was relatively healthy, with a BMI of 25 kg/m^2^ and denied any current or previous smoking history. Lastly, she had already received general anesthesia for a prior parotidectomy, which proceeded without complication. Collectively, the patient did not have any risk factors for TBM, which prevented consideration of TBM on the initial differential as she was decompensating postoperatively.

This patient was fortunate to achieve a positive outcome, but the potential for permanent damage and even death cannot be understated. Vorakunthada et al. reported a similar case of unexpected TBM in a 26-year-old male following extubation [8]. This patient’s dyspnea did not resolve spontaneously, requiring multiple tracheal balloon dilations, cryotherapy, and referral for potential tracheal stenting [8]. Key differences in this case were the patient’s obesity and the extubation following a 14-day ICU admission for staphylococcal sepsis [8].

Our case provides evidence that TBM should be considered in a patient who cannot be intubated and is retaining carbon dioxide despite hyperventilating, even in the absence of risk factors. When providing anesthesia to these patients, the placement of an ET tube is favorable due to the guaranteed luminal patency it provides. However, in many cases, ET tube placement may not be achievable secondary to tracheal collapse. In our patient, we could maintain ventilation with LMA using PEEP. It has been proven that PEEP is therapeutic in patients with TBM due to the intraluminal pressure preventing airway collapse [9]. Alaws et al. reported a case of severe TBM that was successfully stabilized with PEEP, securing enough time to evaluate permanent tracheal stenting candidacy [10]. The sudden ability to ventilate a patient with an LMA with PEEP in whom an ET tube could not be placed should also increase suspicion for potential TBM. Regardless, it is crucial to consider TBM as a potential source of hypoxia in a surgical patient in whom more likely hypoxic etiologies have been ruled out. Schwartz et al. reported a case of TBM in a 73-year-old woman with acute-onset respiratory distress whose alveolar-arterial gradient and modified Well’s score were suggestive of an acute pulmonary embolism [11]. The collapse of the trachea was noticed on contrast-enhanced CT, providing an incidental diagnosis of TBM [11]. A key difference in this case that should be noted is that the patient possessed a history of COPD and pulmonary wedge resection, providing sources of chronic airway trauma [11].

The precise mechanism of action of secondary TBM is poorly understood, especially in patients who do not exhibit any identified risk factors. Extensive postoperative questioning revealed that the patient is an avid singer. There is no formal data suggesting that repetitive singing is a risk factor for TBM; however, it is the only potential source of chronic airway trauma identified.

## Conclusions

Spontaneous TBM is a rare but serious complication of general anesthesia and should always be considered when other more common etiologies of carbon dioxide retention have been ruled out. The tracheal collapse commonly prevents ET tube placement, in which case an LMA with PEEP should be attempted. After stabilization, patients can undergo direct bronchoscopy for confirmation of diagnosis and be allowed to slowly emerge from anesthesia, ensuring the return of full airway protection by sequentially decreasing PEEP before extubation. The key to successful management of spontaneous postoperative TBM is early detection and quick action toward returning ventilation.
